# Modulation of Caspase Activity Regulates Skeletal Muscle Regeneration and Function in Response to Vasopressin and Tumor Necrosis Factor

**DOI:** 10.1371/journal.pone.0005570

**Published:** 2009-05-18

**Authors:** Viviana Moresi, Gisela Garcia-Alvarez, Alessandro Pristerà, Emanuele Rizzuto, Maria C. Albertini, Marco Rocchi, Giovanna Marazzi, David Sassoon, Sergio Adamo, Dario Coletti

**Affiliations:** 1 Department of Histology and Medical Embryology and Interuniversity Institute of Myology, Sapienza University of Rome, Rome, Italy; 2 Myology Group UMR 787, Faculté de Médecine Pitié-Salpétrière, INSERM 787, Paris, France; 3 Institute of Biological Chemistry, University of Urbino, Urbino, Italy; 4 Institute of Biomathematics, University of Urbino, Urbino, Italy; New Mexico State University, United States of America

## Abstract

Muscle homeostasis involves *de novo* myogenesis, as observed in conditions of acute or chronic muscle damage. Tumor Necrosis Factor (TNF) triggers skeletal muscle wasting in several pathological conditions and inhibits muscle regeneration. We show that intramuscular treatment with the myogenic factor Arg^8^-vasopressin (AVP) enhanced skeletal muscle regeneration and rescued the inhibitory effects of TNF on muscle regeneration. The functional analysis of regenerating muscle performance following TNF or AVP treatments revealed that these factors exerted opposite effects on muscle function. Principal component analysis showed that TNF and AVP mainly affect muscle tetanic force and fatigue. Importantly, AVP counteracted the effects of TNF on muscle function when delivered in combination with the latter. Muscle regeneration is, at least in part, regulated by caspase activation, and AVP abrogated TNF-dependent caspase activation. The contrasting effects of AVP and TNF *in vivo* are recapitulated in myogenic cell cultures, which express both PW1, a caspase activator, and Hsp70, a caspase inhibitor. We identified PW1 as a potential Hsp70 partner by screening for proteins interacting with PW1. Hsp70 and PW1 co-immunoprecipitated and co-localized in muscle cells. *In vivo* Hsp70 protein level was upregulated by AVP, and Hsp70 overexpression counteracted the TNF block of muscle regeneration. Our results show that AVP counteracts the effects of TNF through cross-talk at the Hsp70 level. Therefore, muscle regeneration, both in the absence and in the presence of cytokines may be enhanced by increasing Hsp70 expression.

## Introduction

The maintenance of regenerative capacity through recruitment or activation of resident stem cells is important for skeletal muscle recovery following injury or disuse [1]–[3]. Loss of regenerative potential is associated with numerous pathological conditions, including dystrophy and cachexia [4]. Cytokines play an important role both in eliciting muscle wasting and in blocking muscle regeneration [5], [6]. In particular, tumor necrosis factor-α (henceforth referred to as TNF, in agreement with Clark [7]) is a principal cytokine involved in the pathogenesis of muscular dystrophy and other disease states such as cachexia [8]–[10]. Prolonged exposure to TNF is known to block myogenic cell differentiation and muscle regeneration [6], [11]. This occurs, at least in part, through non-apoptotic caspase activation in myogenic cells *in vitro*, which is also observed in interstitial stem cells in the regenerating muscle following focal injury [6]. Caspase inhibitors rescue muscle differentiation *in vitro* as well as muscle regeneration in the presence of TNF, thereby showing that caspase activity is required to mediate the effects of TNF. PW1 is an effector of p53 cell death pathways and mediates Bax translocation to the mitochondria [12]. PW1 and p53 are also jointly involved in mediating cachexia [13]. PW1 is expressed in skeletal muscle throughout development, in cultures of both myogenic cell lines and primary cells as well as in the regenerating muscle [6], [11], [14]. PW1 is responsible for the recruitment of caspase-dependent pathways that inhibit muscle differentiation *in vitro* as well as muscle regeneration [6], [11], [12], [15].

A key regulatory event of the caspase cascade is the association of cytochrome c and apoptotic-protease-activating factor 1 (Apaf-1). Following Bax translocation to the mitochondrial membrane, Apaf-1 is released into the cytosol and initiates the caspase cascade, with the activation of the constitutively expressed procaspase-9 [16]. It has been demonstrated that the inducible heat shock protein Hsp70 regulates caspase activation by directly interacting with Apaf-1, and thereby deters procaspase-9 binding to Apaf-1 for its activation [17]. Hsp70 has been reported to protect skeletal muscle against cryolesion and age-related dysfunction [18], [19]. A more recent study showed that Hsp70 overexpression prevents muscle atrophy [20], thereby extending the beneficial effects of Hsp70 on muscle to the inhibition of protein catabolism through the repression of the transcriptional activities of NF-kB and Foxo3a [20], two factors that induce muscle wasting [21], [22].

Our group has shown that the neurohypophyseal nonapeptide Arg^8^-Vasopressin (AVP) positively regulates myogenic differentiation [23], [24]. In myogenic cells, AVP activates both the calcineurin and CaMK pathways [25]–[27]. Furthermore, AVP removes inhibitory signals, such as elevated cAMP levels, in the early phases of differentiation [28]. We also showed that AVP evoked PLD-mediated cytoskeleton remodeling, which enhances cell-cell fusion during muscle differentiation [29]. AVP, which is physiologically present in the plasma, induces differentiation in serum-free myogenic cell cultures and positively interacts with IGFs to promote muscle cell differentiation through upregulation of Myf5 and myogenin [23]. A physiological role for AVP in skeletal muscle is suggested by the expression of the AVP receptor (V1aR) in human skeletal muscle [30], [31] and of the oxytocin receptor (also a AVP target) in cultured human myoblasts [32]. We have observed upregulation of V1aR expression upon muscle regeneration (manuscript in preparation). An increase in circulating AVP levels during muscular activity has been reported for different animal species, including man [33]–[35]. However, the effects of AVP on skeletal muscle *in vivo* have yet to be fully characterized.

Currently, the only known way to counteract the effects of TNF on skeletal muscle *in vivo* is by competing with TNF using immunological approaches [36]. Nonetheless, we have reported that *in vitro* exposure to a static magnetic field counteracts TNF inhibition of muscle differentiation [37], which suggests that it is possible to override the negative effects of TNF on myogenesis. Here we report for the first time that AVP rescues TNF inhibition of muscle differentiation/regeneration *in vitro* and *in vivo*. The *in vivo* application of our findings is of particular relevance since AVP treatment significantly enhances the performance of regenerating muscles. Muscle cells express both PW1 and Hsp70, and we show that these two factors interact. We also show that TNF activates caspases *in vivo* by downregulating Hsp70 without affecting PW1 expression. By contrast, AVP increases Hsp70 expression in muscle. Hsp70 overexpression inhibits caspase activation and rescues muscle regeneration in the presence of TNF. Taken together, our results highlight the existence of cross-talk between TNF and AVP-dependent pathways at the Hsp70 level that may be exploited for gene or pharmacological approaches aimed at enhancing muscle regeneration.

## Results

### AVP counteracts TNF-mediated inhibition of myogenic differentiation *in vitro*

**Figure 1:**
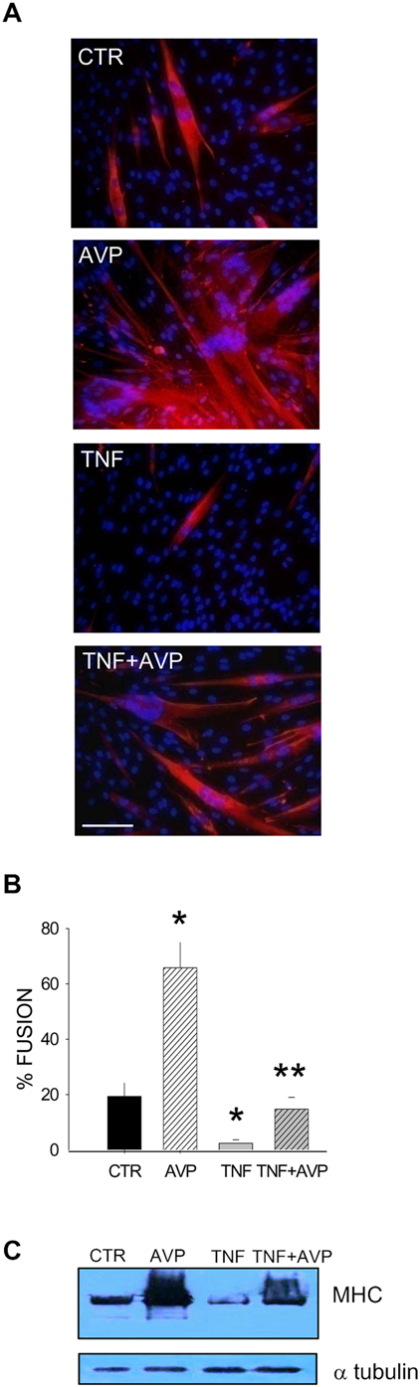
TNF and AVP differentially regulate myogenic differentiation *in vitro*. A) The L6 cell line was induced to differentiate in the continuous presence of PBS (*CTR*), AVP, TNF, or TNF and AVP combined (*TNF+AVP*). Myosin (red) was immunostained to assess cell differentiation. Nuclei were detected with DAPI (blue). Scale bar = 100 micron. B) Quantitative analysis of myogenic differentiation (*% FUSION*) of L6 cells. ** = p<0.04 vs TNF; * = p<0.05 vs CTR, by Student's *t* test. Data are the means±SEM of values from three independent experiments performed in triplicate. C) Myosin (*MHC*) and α-tubulin levels detected by western blot in cells treated as described above. TNF blocks whereas AVP promotes myogenic differentiation. In the presence of TNF, AVP rescues myogenic differentiation to the level of the control. Data are representative of three independent experiments.

In order to test the ability of AVP to counteract TNF-mediated inhibition of muscle differentiation, we induced differentiation of the myogenic cell line L6 in the absence or in the presence of TNF, AVP, or TNF and AVP combined. When cultured in the presence of low serum levels L6 cells displayed the ability to form multinucleated, myosin positive myotubes. Figure 1 shows that TNF and AVP had opposite effects on this phenomenon, as can be seen from both the fusion index and WB analysis for myosin heavy chain content. However, AVP combined with TNF rescued myogenic differentiation to levels similar to those of the control (Fig. 1A, B and C).

### Hsp70 binds to PW1 without affecting its expression

PW1 is a pivotal mediator of the pleiotropic effects of TNF and mediates several pathways, including caspase and NF-kB activation [11], [38], [39]. In an attempt to find novel PW1 partners, PW1 was immunoprecipitated from several cell lines, and PW1 immunoprecipitation products were subjected to two-dimensional electrophoretic analysis and liquid chromatography coupled with tandem mass spectrometry (LC-MS/MS). This approach highlighted several potential interactions, including the binding of PW1 to Hsp70 (Supplemental Fig. S1). Indeed, LC-MS/MS revealed that the majority of the spots in 2D-gels consisted of Hsp70 peptides corresponding to fragments of Hsp70 previously isolated as co-immunoprecipitation product. These evidence occurred in muscle cells, which endogenously express PW1, and in non-muscle cells, where PW1 expression was ectopically driven. This pointed out Hsp70 as a potential candidate to form complexes with PW1 and prompted us to further investigate PW1 and Hsp70 expression and interaction.

**Figure 2:**
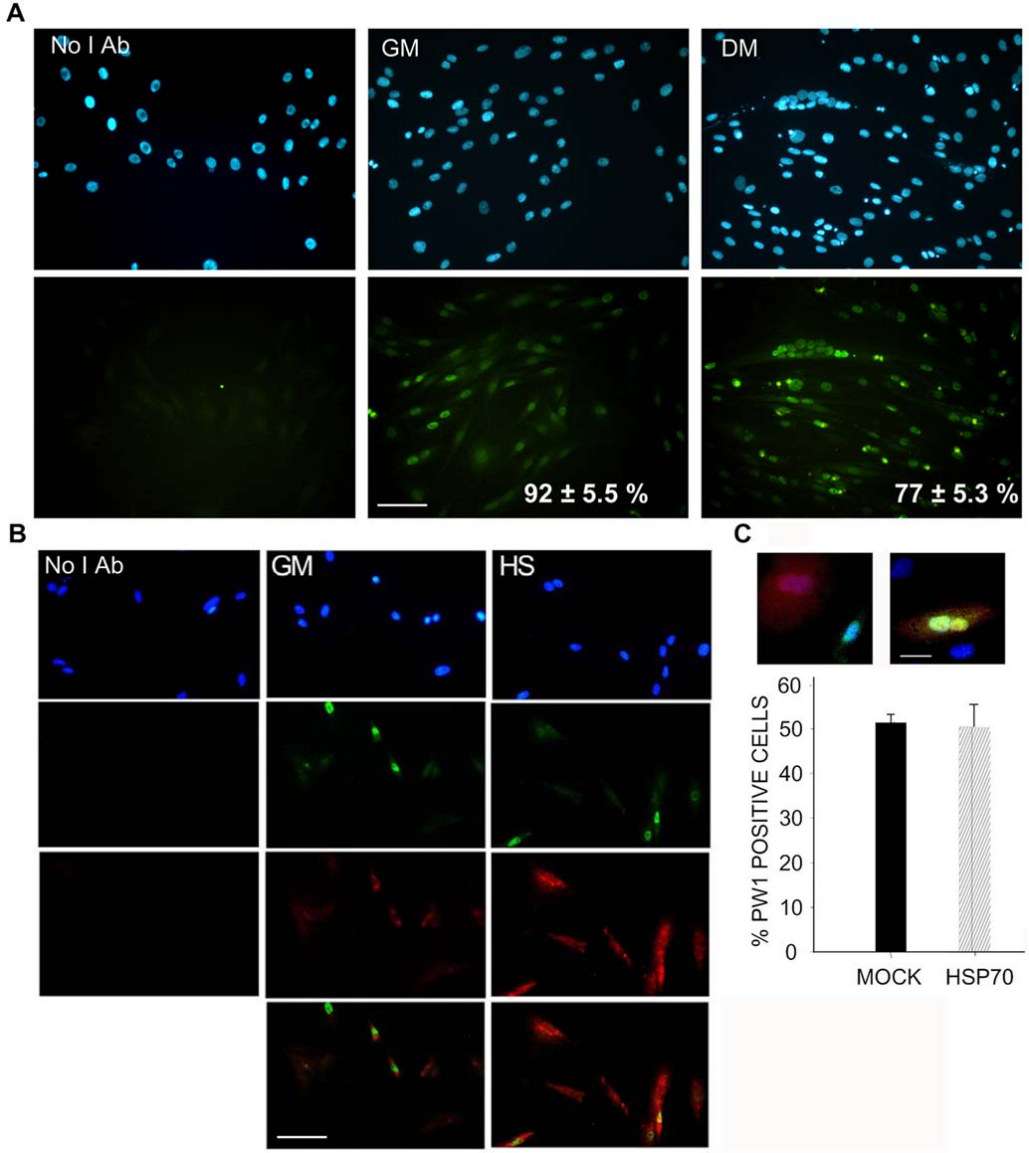
Hsp70 and PW1 are coexpressed and colocalize in L6 cells. A) Immunofluorescence analysis of PW1 expression in L6 cells in growth medium (*GM*) and following 5 d of differentiation (*DM*). PW1 (green) displays nuclear and cytoplasmic localizations in both conditions. DAPI-stained nuclei are blue. Panels on the left represent a negative control incubated without primary Ab (*No I Ab*). Embedded in the panels is the mean percentage of PW1-expressing cells±SEM, resulting from three independent experiments performed in triplicate. Scale bar = 100 micron. B) Immunofluorescence analysis of Hsp70 (red) and PW1 (green) expression in L6 cells cultured in *GM* subjected or not subjected to heat shock treatment (*HS*), as described in the Materials and Methods. Nuclei were visualized by DAPI staining (blue). The lowest panels represent merged images. The panels on the left represent a negative control incubated without primary Ab (*No I Ab*). PW1 and Hsp70 are expressed in the same cells, both in basal conditions and following heat shock treatment, which upregulates Hsp70 expression. PW1 and Hsp70 colocalize in both the cell nucleus and cytoplasm. Data are representative of six independent experiments. Scale bar = 100 micron. C) Immunofluorescence analysis of PW1 expression in L6 cells overexpressing Hsp70. PW1 staining (green) was performed on cells transfected with an expression vector for Hsp70. Transfected cells were identified by coexpression of red fluorescent protein (RFP, red). Nuclei are counterstained with DAPI (blue) and tricolour, merged images are shown. All the possible combinations were observed, with cells expressing or not expressing PW1 in the presence of RFP and Hsp70 expression. The percentage of PW1 positive cells among the cells transfected with either pCDNA and RFP or Hsp70 and RFP was evaluated in 10 randomly chosen fields for each sample. Shown is the mean±SEM of three replicate experiments. Hsp70 and PW1 are both expressed in muscle cells and Hsp70 expression levels do not affect PW1 expression.

While PW1 expression is widespread in both myogenic cell lines and primary cell cultures as well as in muscle stem cells *in vivo*, its expression in the myogenic cell line L6 has not been reported to date. We observed that PW1 is expressed in the vast majority of L6 cells both in growth medium and upon differentiation (Fig. 2A). We noted that PW1 has a double nuclear and cytoplasmic localization in both single cells and myotubes. We also demonstrated that Hsp70 is constitutively expressed in L6 cells and its expression is upregulated by heat shock (Fig. 2B). PW1 is coexpressed with Hsp70 in myogenic cells both in the basal condition and upon heat shock treatment (Fig. 2B), which supports previous data pointing to a possible interaction. To verify whether increased Hsp70 expression affects PW1 expression or localization, we overexpressed Hsp70 in L6 cells and found that this has no effect either on the percentage of cells expressing PW1 or on its localization (Fig. 2C).

**Figure 3:**
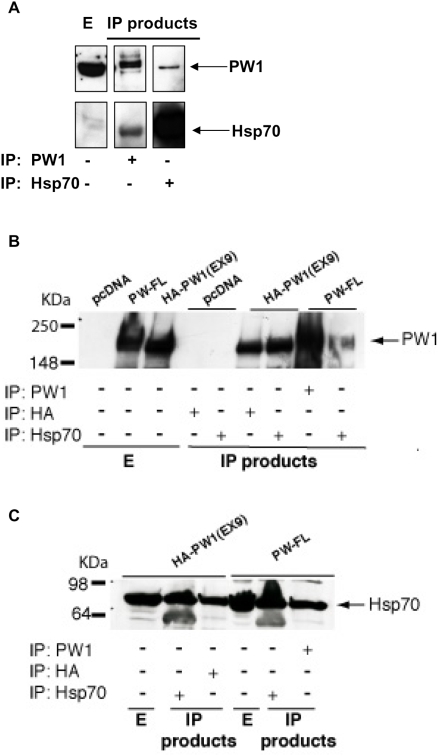
Hsp70 and PW1 physically associate. A) Co-immunoprecipitation of PW1 and Hsp70 in myogenic cells. Equal amounts of L6 whole cell extracts (*E*) were subjected to immunoprecipitation (*IP*) of endogenous PW1 or endogenous Hsp70 proteins using an anti-PW1 antibody or an anti-Hsp70 antibody, respectively. The presence of PW1 and Hsp70 was assessed by Western blot on whole cell extracts and on the immunoprecipitated products. B) Co-immunoprecipitation of Hsp70 and ectopically expressed PW1. HEK293 cells were transiently co-transfected with an expression vector containing the HA-tagged exon 9 region of PW1 (*HA-PW1 EX9*), a full-length PW1 construct (*PW1 FL*) or the empty vector (*pcDNA*), by the calcium phosphate method. 48 h after transfection, equal amounts of whole cell extract (500 µg) were subjected to immunoprecipitation of PW1 or Hsp70 as indicated, by using a mouse monoclonal antibody against HA tag or against Hsp70, or a polyclonal antibody against PW1 (*IP*). The pellets of the immunoprecipitation or an aliquot of the whole extract were analyzed by Western blot using an anti-PW1 antibody. In C the reciprocal experiment is shown, where HEK293 cells were transiently co-transfected with an expression vector for HA-tagged PW1 (*HA-PW1 EX9*), or a full-length PW1 construct (*PW1 FL*). Cell extracts were subjected to immunoprecipitation of PW1 or Hsp70 as indicated, by using a mouse monoclonal antibody against HA tag or against Hsp70, or a polyclonal antibody against PW1 (*IP*). The pellets of the immunoprecipitation or an aliquot of the whole extract were analyzed by Western blot using an anti-Hsp70 antibody. A physical interaction between PW1 and Hsp70 is demonstrated both for the endogenous proteins in muscle cells and when PW1 is ectopically expressed in non-muscle cells. Data are representative of at least three independent experiments.

Co-immunoprecipitation experiments of endogenous PW1 and Hsp70 were performed and confirmed that L6 cells express both factors. In particular, we showed the presence of PW1-Hsp70 complexes by immunoprecipitating cell extracts with an anti-PW1 Ab and blotting with an anti-Hsp70 Ab, or by inverting this procedure (Fig. 3A). To further demonstrate the interaction between Hsp70 and PW1, the latter was ectopically expressed in 293 cells by transient transfection; cotransfection with an expression vector for ß-galactosidase was used to monitor transfection efficiency (data not shown). To overexpress PW1, two different constructs were used: one contained the HA region as a tag followed by the sequence corresponding to its exon 9, while the other contained the full length form of PW1. WB analysis of cell extracts revealed robust PW1 expression following expression of both constructs (Fig. 3B). Under these conditions, both an anti-HA Ab and an anti-HSP70 Ab immunoprecipitated PW1 (Fig. 3B). Immunoprecipitation was specific, as demonstrated by the fact that no PW1 was recovered when an empty vector was expressed in the cells. In addition, we found Hsp70 in a complex immunoprecipitated with an anti-HA Ab as well as with an anti-PW1 Ab in cells overexpressing PW1 (Fig. 3C). These findings indicate Hsp70-PW1 interaction.

### Hsp70 reduces TNF-dependent caspase activity

**Figure 4:**
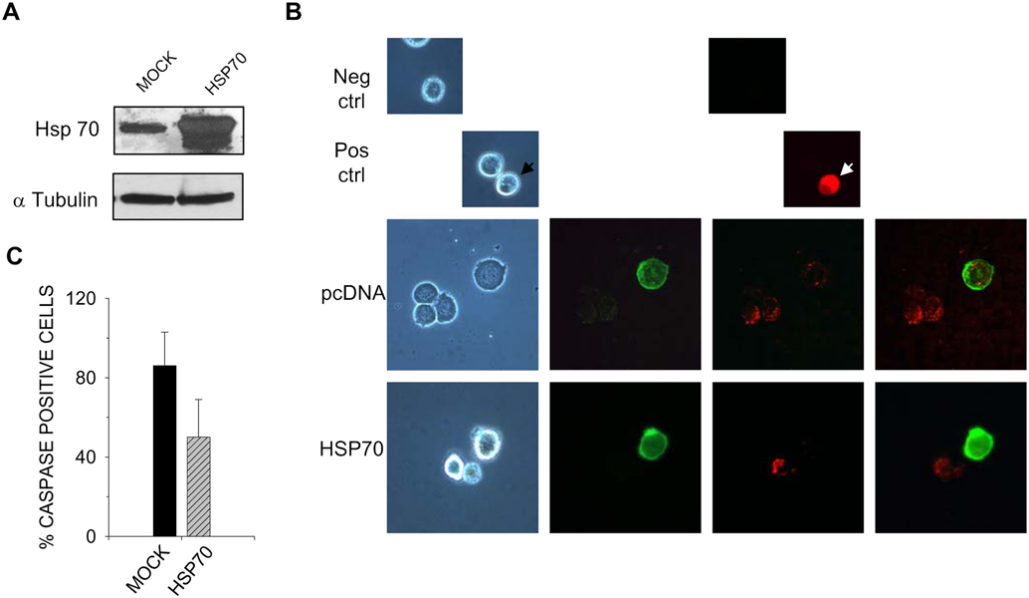
Hsp70 overexpression reduces TNF-mediated caspase activation. A) L6 cells were transfected with SNAP-GFP and an excess of Hsp70 expression vector or empty vector. Overexpression of Hsp70 was assessed by Western Blot analysis of cell lysates normalized for tubulin expression. The cells, transfected as indicated, were treated for 16 h with TNF, and floating and adherent cells (detached by trypsin) were pooled. B) Caspase activity (red) was detected with CaspGLOW while transfected cells were detected by the expression of SNAP-GFP (green). Puromycin-treated, apoptotic cells incubated or not incubated with CaspGLOW were used as a positive (Pos ctrl) and negative (Neg ctrl) control for the caspase assay, respectively. From left to right, the panels show phase contrast, green channel, red channel and merged images (the latter with increased contrast to highlight both signals). Only the apoptotic cell (*arrowhead*), characterized by a condensed nucleus, shows a bright fluorescence due to intense caspase activation. TNF-treated cells display a weaker fluorescence than apoptotic cells. C) The percentage of mock transfected cells that displayed caspase activity was evaluated by counting 10 randomly chosen fields in triplicate experiments. Shown is the mean±SEM of values from three independent experiments. Hsp70 overexpression reduces the percentage of cells displaying active caspases in the presence of TNF.

TNF induced caspase activation in L6 cells. Given the well-established Hsp70 inhibitory effects on caspase cascade activation, we tested whether Hsp70 affected TNF-dependent caspase activation in L6 cells. We overexpressed Hsp70 in these cells and verified the increased levels of this protein by WB analysis (Fig. 4A) and, on a single cells basis, by cotransfection with an expression vector for green fluorescence protein (SNAP-GFP) (Fig. 4B). The latter allowed us to follow the effects of Hsp70 overexpression on TNF-induced caspase activity, highlighted by means of a red-fluorescence caspase substratum (Fig. 4B). Most (86%) of the mock transfected, TNF-treated L6 cells displayed caspase activation, whereas only 50% of the Hsp70 expressing cells displayed caspase activation in the presence of TNF (Fig. 4C). As controls for the caspase assay, we used puromycin-treated cells incubated, respectively, with or without the red-fluorescence caspase substratum (Fig. 4B, small, upper panels).

### AVP counteracts the negative effects of TNF on muscle regeneration

**Figure 5:**
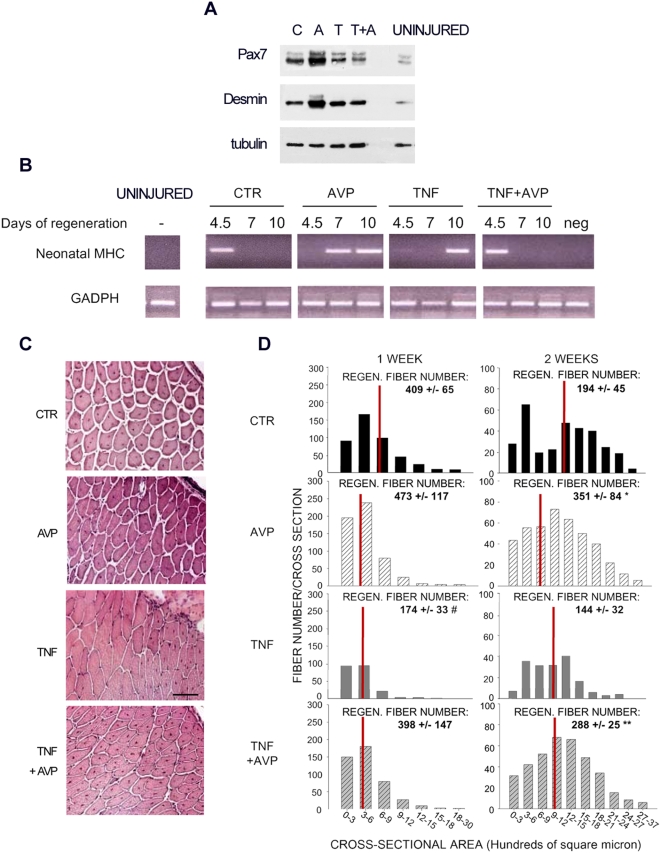
AVP counteracts TNF inhibition of muscle regeneration. A) WB analysis on extracts from adult (*UNINJURED*) and regenerating *Tibialis anterior* injected with PBS (*C*), AVP (*A*), , TNF (*T*) or TNF and AVP combined (*T+A*) and analyzed at 4.5 days following injury. The levels of Pax7 and desmin proteins were used as markers of satellite cell activation, while the levels of tubulin were the loading control. AVP increases satellite cell activation, while TNF does not affect this phenomenon and, in combination with AVP, allows satellite cell activation similar to the control. Data are representative of at least three independent experiments.B) RT-PCR from adult (*UNINJURED*) and regenerating *Tibialis anterior* injected with PBS (*CTRL*), TNF (*TNF*), AVP (*AVP*), or TNF and AVP combined (*TA*) and analyzed at 4.5, 7 and 10 days following injury. Neonatal myosin heavy chain expression was monitored, normalized by the GADPH expression. No RNA was reverse-transcripted in the negative control (*neg*). Data are representative of three independent experiments C) Injured *Tibialis anterior* injected with PBS (*CTR*), TNF, AVP, or TNF and AVP combined (*TNF+AVP*) 2 and 4 days following injury, and analyzed 1 or 2 weeks after injury. Representative H&E-stained muscle cross cryosections showing regenerating fibers, after 2 weeks of regeneration, characterized by the presence of centrally located nuclei, 2 weeks after injury. Insets, higher magnification. Scale bar = 100 micron. D) Histograms showing the distribution of the regenerating fiber cross sectional areas (*FIBER NUMBER/CROSS SECTION*) in different size classes (*CROSS-SECTIONAL AREA*), 1 week (left column) and 2 weeks (right column) after injury. The median value is shown as a red bar. The total number of regenerating fibers for muscle cross section (*REGEN. FIBER NUMBER*) is shown on top of each panel. Data are the means±SEM of values from at least three independent experiments performed in triplicate (9<n<14 for each experimental condition). ANOVA showed a highly significant effect of treatments on muscle regeneration 2 weeks after injury. LSD was used as a *post hoc* test. # = p<0.05 vs CTR; * = p<0.05 vs CTR or vs TNF; ** = p<0.05 vs TNF. The following phenomena are apparent at 2 weeks of regeneration: AVP enhances whereas TNF reduces muscle regeneration; AVP treatment counteracts the negative effects of TNF.

We have reported that TNF injection into regenerating muscle hampers muscle regeneration following focal injury [6]. We demonstrated the ability of AVP to override the negative effects of TNF on myogenic differentiation *in vitro*. We therefore extended our analysis to injured muscles, treated with a combination of AVP and TNF, in order to evaluate the ability of AVP to rescue regeneration in the presence of TNF. We analyzed the effects of these treatments on the initial regenerative response, by WB evaluation of satellite cell activation markers, namely Pax7 and desmin. We found that at day 4.5 of regeneration AVP greatly increased the levels of both Pax7 and desmin, as compared to the control, while TNF did not affect the levels of these proteins (Fig. 5A); the combination of AVP and TNF determined lower levels of Pax7 but not of desmin, as compared to the control, and lower levels of both markers if compared to AVP treatment (Fig. 5A). The expression kinetics of neonatal myosin heavy chain (neoMHC), a marker expressed by nascent regenerating fiber, showed, when compared with the control, that AVP extends the expression of this marker, TNF delays neoMHC expression, while the combination of AVP and TNF yields a peak of neoMHC no later that 4.5 days following injury, i.e. overlapping that of the control (Fig. 5B).

To assess regeneration at the morphological level, we analyzed the fibers with centrally located nuclei. For this purpose, we counted the number of regenerating fibers per cross-section and we measured the regenerating fiber cross-sectional area (Fig. 5C and D). One-way Anova analysis showed that intramuscular treatments effected the number of regenerating fibers by a quasi-significant level after one week of regeneration (df:3,19; F = 3.137, p = 0.05). Anova then showed that treatments significantly increased the number of regenerating fibers after two weeks of regeneration (df:3,17; F = 4.657, p = 0.02), thus suggesting that treatments required time to fully exert their effects on muscle regeneration. As shown in Figures 5C and D, TNF and AVP exerted opposite effects on muscle regeneration, these effects becoming particularly evident after two weeks of regeneration. At this time point, AVP treatment determined a significant increase in the number and size of regenerating fibers if compared with both control and TNF-treated muscles. In addition, AVP fully rescued the TNF-mediated reduction in the number of regenerating fibers, following which fiber size distribution mirrored that obtained upon treatment with AVP alone (Fig. 5D).

Overall, these data suggest that AVP and TNF affect muscle regeneration in different ways, and that it may be possible to exploit AVP to enhance myogenesis *in vivo* and counteract the negative effects of TNF on regeneration.

### Muscle performance mirrors the extent of muscle regeneration

**Figure 6:**
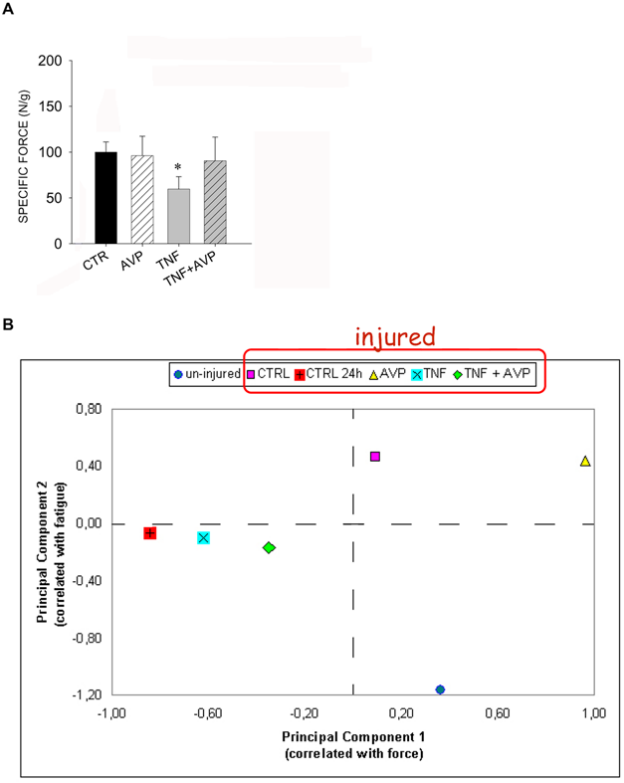
AVP counteracts TNF inhibition of muscle function. A) AVP and TNF differentially affect muscle performance. The *Tibialis anterior* muscle was subjected to freeze injury, injected with PBS (*CTR*), AVP, TNF, or TNF and AVP combined (*TNF+AVP*) 2 and 4 days later, and analyzed 1 week following injury. One week after injury, the TNF-treated muscle generates a tetanic force that is significantly lower than that achieved with all other treatments. Data obtained from 6<n<7, for each experimental condition, are shown as mean±SEM; * p<0.05 vs. CTR by Student's t test. B) Principal Components Analysis (PCA) was performed using the multiple parameters from the functional data sets obtained both 1 and 2 weeks following injury, as described in the Materials and Methods. One example of a data set included in the PCA are the specific force recordings described in A. The centroids represent the mean values obtained from single measurements on the muscles of each of the 6 treatment groups (uninjured; CTRL 24 h; CTRL; AVP; TNF; AVP+TNF). The centroids were plotted in the bidimensional space defined by the 1^st^ and 2^nd^ principal component functions shown in Table 3. The differences in behaviour between the injured muscles and uninjured muscles vary significantly depending on the treatments: the TNF-treated muscle exhibits the greatest difference from uninjured muscle and is closest to freshly injured muscle (CTRL 24 h, i.e. a time gap that is not sufficient for functional recovery to occur), which indicates that TNF hampers functional recovery of damaged muscle, whereas AVP rescues this phenomenon.

We performed a functional analysis of regenerating muscles treated as described above to evaluate whether differential regeneration altered muscle performance. An isometric experimental protocol was applied to characterize the mechanical properties of uninjured and of regenerating muscles treated with TNF, AVP, or TNF and AVP combined. An explorative analysis performed by Student's *t* test on the functional properties of muscle pointed to the existence of a significant difference between the mean tetanic force and the mean specific force of TNF-treated muscles, compared with the control regenerating muscle, after 1 week of regeneration (Table 1 and Fig. 6A), suggesting that TNF hampered muscle functional recovery after injury (Table 1). By contrast, AVP treatment rescued muscle performance in the presence of TNF (Table 1 and Fig. 6A). No significant differences between treatments were observed in the fatigue time of regenerating muscle one week after injury (Table 1).

**Table 1:** Functional analysis of skeletal muscle after one week of regeneration.

Parameters	Treatments			
	CTR	TNF	AVP	TNF+AVP
Force (% of CTR)	**100.0**±3.3	59.1±12.9*	88.6±22.3	93.1±20.0
Specific Force (% of CTR)	**100.0**±11.1	50.7±7.6*	96.1±21.2	90.5±25.9
Fatigue time (sec)	18.8±1.14	19.0±0.4	18.8±1.3	18.4±0.7

Injured *Tibialis anterior* muscles were injected with PBS (*CTR*), *TNF*, *AVP* or TNF and AVP combined (*TNF+AVP*) 2 and 4 days after freeze injury, and analyzed after one week of regeneration. Data obtained from 6<n<7, for each experimental condition, are shown as mean±SEM.

* p<0.05 vs. CTR by Student's *t* test.

Since the morphological effects exerted by TNF and AVP on muscle regeneration peaked two weeks after injury, we extended the muscle functional analysis to this time point of regeneration. We noted that the average specific force of regenerating, control muscle was 87±10.3 mN and 90±4.9 mN at one and two weeks following injury, respectively. The two values are not significantly different. As Multiple Anova (Manova) analysis showed that the treatments described above affected muscle performance (P<0.0005 by Hotelling's Trace test), we decided to further investigate which muscle functional parameters were significantly affected in the different conditions. One-way Anova analysis, performed on muscles two weeks after injury, revealed significant differences in tetanic and specific force as well as in fatigue time between the treated muscles (with treatments being: PBS, TNF, AVP and TNF+AVP; df:5; F:4.486; p = 0.002 for tetanic force; df:5; F:4.489; p = 0.002 for specific force; df:5; F:3.006; p = 0.018 for fatigue time; total df:63). As summarized in Table 2, we statistically investigated which muscle functional properties were affected most. This *post hoc* analysis showed that the specific and tetanic force of adult, uninjured muscles was significantly different from that of regenerating muscle (p<0.05 by LSD test), with the exception of the AVP-treated muscles. In particular, 24 hours after injury, the tetanic and specific muscle tetanic force was significantly lower (by more than 50%) than that of adult uninjured muscles (p<0.005 at LSD test); this deficit was partially recovered in control conditions and, though to a lesser extent, upon TNF treatment, which reduced both tetanic and specific force by about 10% if compared with controls; AVP-treated muscle exhibited a significant increase in tetanic and specific force (approx. 34%) if compared with regenerating control muscle (p<0.05 by LSD test); AVP combined with TNF yielded a muscle tetanic force comparable to that of TNF-treated muscle (Table 2). However, the force deficit observed in TNF-treated muscle was accompanied by a more rapid onset of fatigue (p<0.05 at LSD test), which was reversed by AVP treatment (Table 2). Thus, TNF worsened the performance of regenerating muscle, AVP enhanced performance, whereas combined TNF and AVP treatment yielded muscle functional output comparable to that of control muscles.

**Table 2:** Functional analysis of skeletal muscles after two weeks of regeneration.

Parameters	Treatments					
	UNINJURED	CTR 24 h	CTR	TNF	AVP	TNF+AVP
Force (% of CTR)	143.9±35.2*	68.5±11.2°	**100.0**±9.0	92.2±15.0	134.0±7.3#	87.6±6.7
Specific Force (% of CTR)	129.1±37.0*	53.2±16.5°	**100.0**±8.9	87.1±14.9	134.2±8.5#	87.2±26.2
Fatigue time (sec)	15.63±0.01	15.8±0.2	21.7±2.0	17.0±2.0#	20.6±2.3	20.1±1.0

Injured *Tibialis anterior* muscles were injected with PBS (*CTR*), TNF, AVP or TNF and AVP combined (*TNF+AVP*) 2 and 4 days after freeze injury and analyzed two weeks following injury. Regenerating muscles were compared with uninjured muscles and freshly (24 h following damage) injured muscles. Data obtained from 8<n<17, for each experimental condition, are shown as mean±SEM.

* p<0.05 vs. CTR, TNF or TNF+AVP and p<0.005 vs. CTR 24 h by LSD test.

° p<0.005 vs. UNINJURED and p<0.05 vs. either CTR or AVP by LSD TEST.

# p<0.03 vs. CTR by LSD test.

The beneficial effects of AVP counteracting the negative effects of TNF were less evident after two weeks than after one week of regeneration, probably owing to the partial functional recovery of the injured muscles 2 weeks after injury regardless of the treatment. In order to determine the similarities and differences between the treatments more accurately, we performed a principal components analysis (PCA) both on the afore-mentioned parameters and on additional parameters of muscle performance. PCA highlighted the two parameters that account for most of the variability between the AVP and TNF treatments: muscle force and fatigue (Table 3). In particular, the 1^st^ principal component closely correlated with tetanic force and specific force, while the 2nd principal component closely correlated with the fatigue index and fatigue time (the variables correlated with the two principal components can be identified by the highest score coefficients in the absolute values shown in Table 3). To depict a large set of data, a diagram of the centroids (indicating the mean values of each treatment group) was plotted in a bidimensional space, defined by the first and second principal components (Fig. 6B). The position of a centroid in the graph thus reflects and summarizes the behavior of a group of treated muscles. Indeed, the two principal components varied markedly depending on the different muscle treatments (Fig. 6B). In contrast to injured muscles, the uninjured muscles yielded positive and negative values respectively for the 1^st^ and 2^nd^ principal components (as shown by their position relative to the two zero axes in Figure 6B). The behavior that differed most from that of the freshly injured muscle was that of the uninjured muscle (ctrl 24 h), which yielded 1^st^ and 2^nd^ principal component negative values. CTR and AVP-treated muscle centroids yielded positive 1^st^ and 2^nd^ principal component values, which are indicative of a progression toward normal functional parameters (Fig. 6B). TNF-treated muscle performance resembled that of control muscle 24 hours after injury despite the fact that the TNF-treated muscles were afforded a significant recovery time (two weeks after injury); this points to the persistence of a functional deficit. It is noteworthy that AVP treatment combined with TNF significantly enhanced muscle function, as shown by the shift of the centroid toward the position of the injured and uninjured controls (Fig. 6B).

**Table 3:** Component score coefficient matrix used for Principal Component Analysis.

Variables	1^st^ Principal Component	2^nd^ Principal Component
Tetanic force	0.444	−0.243
Weight of each muscle	−0.019	−0.084
Specific force	0.450	−0.216
Fatigue index	0.154	0.546
Fatigue time	0.249	0.478

Shown are the coefficients by which variables are multiplied to obtain the principal components as described in the Materials and Methods. The variables that correlate with the two principal components (highest score coefficient absolute values) best are tetanic force and specific force for the 1st principal component, and the fatigue index and fatigue time for the 2nd principal component.

Overall, the functional performance yielded by different treatments correlated with the extent of muscle regeneration, thereby highlighting the importance of prompt regeneration for the full recovery of muscle functional efficiency.

### AVP counteracts TNF-mediated caspase activation, affecting Hsp70 but not PW1 expression

**Figure 7:**
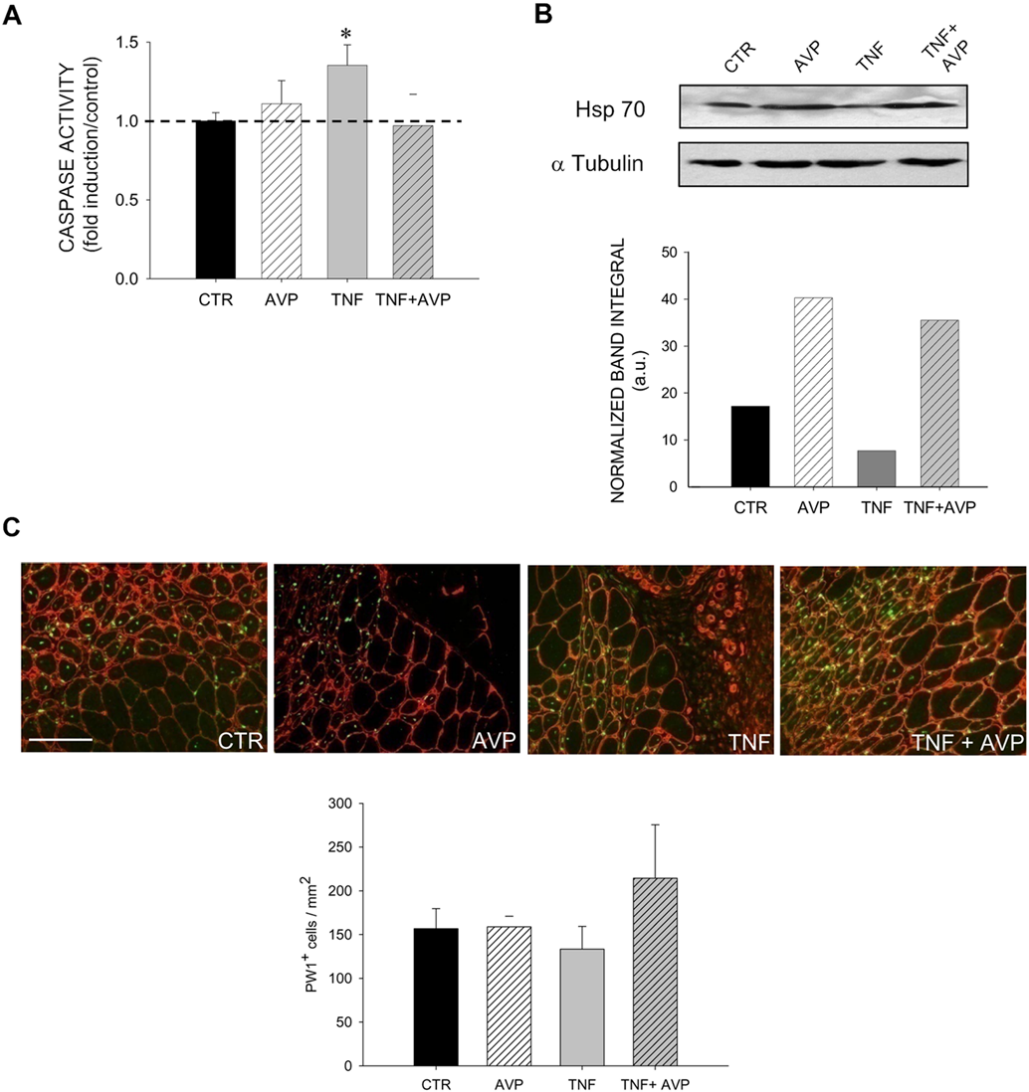
AVP counteracts TNF-mediated caspase activation and modulates Hsp70 but not PW1 expression. The *Tibialis anterior* was injured and injected with PBS (*CTR*), *TNF*, *AVP* or TNF and AVP combined (*TNF+AVP*) 2 and 4 days later. A) Caspase activity was detected in muscle lysates 4.5 days following injury by fluorimetric analysis, as described in the Materials and Methods. Caspase activity was normalized by DNA content and expressed as a fold increase over PBS treated muscles (*CTR*). The results shown are the mean±SEM of five independent experiments performed in triplicate. Student's *t* test: * = p<0.03 vs CTR. TNF significantly increases caspase activity in regenerating muscles, an effect rescued by co-treatment with AVP. B) Western blot analysis on regenerating *Tibialis anterior*, treated as described above, 1 week following injury and relative density plot of Hsp70 bands normalized over tubulin band density. TNF reduces Hsp70 protein levels, whereas AVP increases Hsp70 protein levels in regenerating muscles in the absence or presence of TNF. C) Regenerating *Tibialis anterior* cryosections stained for PW1 (green) and laminin (red) by indirect immunofluorescence 4.5 days after injury. PW1 positive nuclei/mm^2^ of the regenerating area vs treatment were evaluated and plotted. The results shown are the mean±SEM of three independent experiments. Scale bar = 100 micron. No statistically significant difference in the number of PW1 expressing cells was observed.

Given the pivotal role played by caspases in TNF-dependent inhibition of myogenesis *in vitro* and *in vivo*, we investigated the effects of AVP on caspase activity, both in the absence and presence of TNF. By means of fluorimetric assay on regenerating muscle extracts, we showed that AVP did not, unlike TNF which mediated a significant increase in caspase activity, modulate caspase activity (Fig. 7A). Worthy of note is the fact that AVP blocked the TNF-mediated increase in caspase activity when combined with TNF (Fig. 7A).

Given the ability of Hsp70 to inhibit caspase activation (as shown in Figure 4), we measured Hsp70 protein levels in these conditions. We found that AVP and TNF modulate Hsp70 levels positively and negatively, respectively (Fig. 7B). AVP was able to override the effects of TNF on protein levels when the two factors were combined (Fig. 7B).

Immunofluorescence analysis for PW1 and for laminin revealed that PW1 expression is high both in the interstitial space and in the muscle compartment of regenerating muscle, but is not significantly modulated by any treatment (Fig. 7C).

### Hsp70 counteracts TNF-dependent inhibition of regeneration

**Figure 8:**
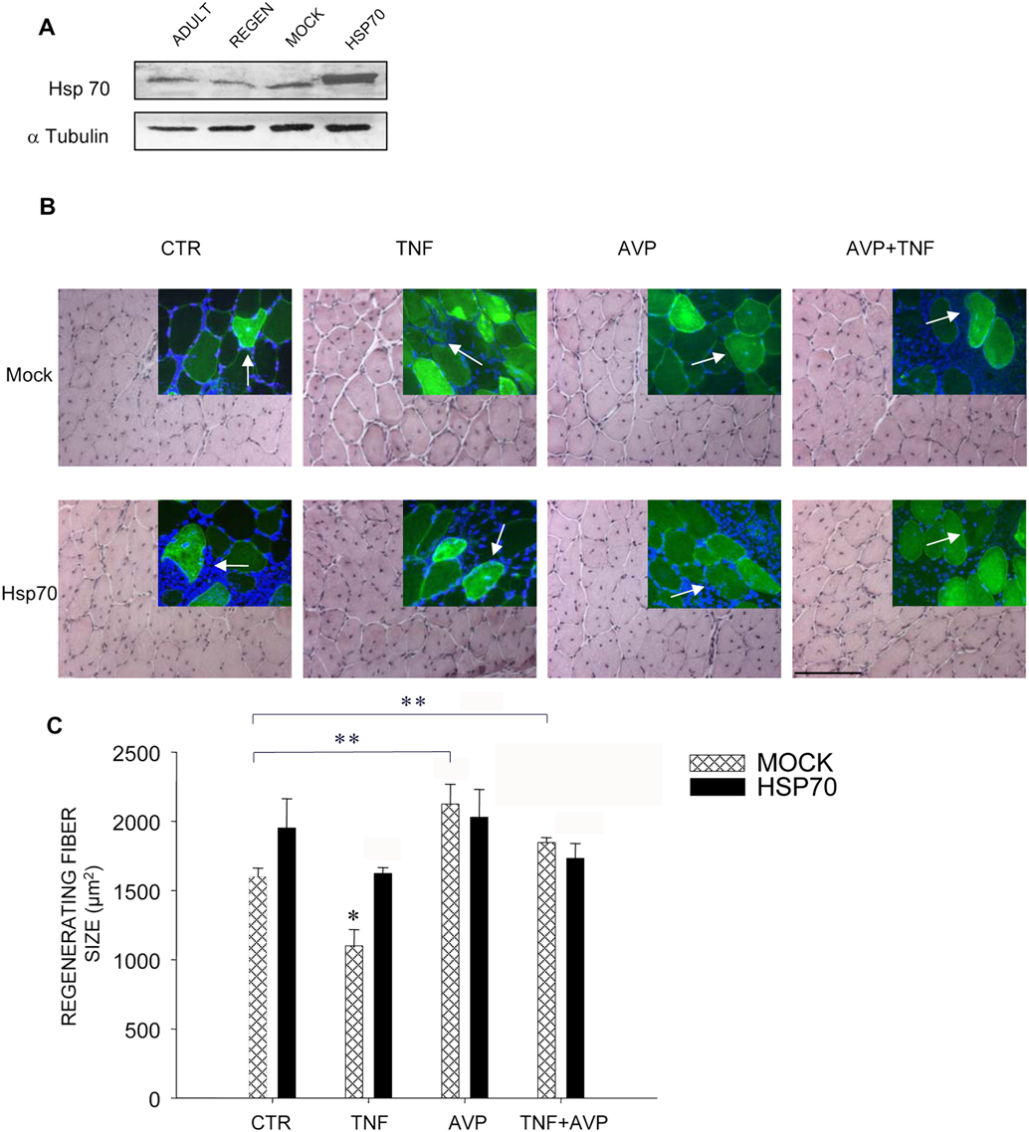
Hsp70 overexpression rescues muscle regenerating fiber size in the presence of TNF. The *Tibialis anterior* was injured and injected with PBS (*CTR*), *TNF*, *AVP* or TNF and AVP combined (*TNF+AVP*) 2 and 4 days later. A) Hsp70 expression by Western blot analysis in adult, uninjured (*ADULT*) and regenerating (*REGEN*) *Tibialis anterior* 1 week following injury; also shown are Hsp70 expression levels in muscles electroporated with an empty (*MOCK*) or an Hsp70 expression vector (*HSP70*) after 4.5 days of regeneration and analyzed after 1 week of regeneration. Hsp70 is not upregulated during regeneration and is enhanced by electroporation-mediated gene delivery. B) The *Tibialis anterior* were injured and injected with PBS (*CTR*), *TNF*, *AVP* or TNF and AVP combined (*TNF+AVP*) 2 and 4 days later. Gene delivery by electroporation of the expression vector for Hsp70 (*HSP70*) or the matching empty vector (*MOCK*) was performed 4.5 days after injury. H&E staining of representative areas is shown. The insets show SNAP-GFP expression (green) in adult and in regenerating fibers (*arrows*), which can be identified by the presence of centrally located nuclei (blue). Scale bar = 100 micron. C) Muscles were analyzed after two weeks of regeneration by measuring the cross-sectional area of the regenerating fibers (*REGENERATING FIBER SIZE*). Data are the means±SEM of values from three independent experiments performed in duplicate. The regenerating fiber size of TNF-treated, MOCK-electroporated samples was significantly different vs. all other conditions tested, such as: PBS-treated, MOCK-electroporated; AVP-treated, MOCK-electroporated; TNF+AVP-treated, MOCK-electroporated; PBS-treated, Hsp70-electroporated; TNF-treated, Hsp70-electroporated; AVP-treated, Hsp70-electroporated; TNF+AVP-treated, Hsp70-electroporated (* = p<0.02 vs. any of the above, by Tukey HSD test). AVP promotes regenerating fiber growth (** = p<0.02 vs MOCK CTR, by Tukey HSD test). Thus, mock electroporated muscles recapitulate the response to treatments observed in non-electroporated muscle. Hsp70 overexpression abrogates TNF-mediated block of regenerating fiber growth, a phenomenon which is not further enhanced by concomitant AVP treatment.

Since protein levels appeared to be inversely correlated to caspase activity in response to TNF and/or AVP, we overexpressed Hsp70 by electroporation-mediated gene delivery in the regenerating muscle to investigate its effects on regeneration. The efficiency of Hsp70 overexpression in electroporated muscles was shown by WB analysis and co-transfection with an expression vector for SNAP-GFP (Fig. 8A and B). In this context, we observed that Hsp70 is expressed at similar levels in adult and regenerating muscle, which is in agreement with a previous report [40]. To analyze the output of Hsp70 overexpression in regenerating muscle treated with AVP and/or TNF, we measured the regenerating fiber cross-sectional area after two weeks of regeneration (Fig. 8B and C). Two-way Anova revealed that both Hsp70 overexpression and the treatments significantly affected the regenerating fiber area (F = 4.77, p = 0.0442 for hsp70 overexpression; F = 14.93, p = 0.0001 for treatments). Anova also showed that Hsp70 overexpression *in vivo* interacted with treatments, thereby affecting the regenerating fiber size (F = 4.44, p = 0.0188 for interaction Hsp70 x treatments). In particular, mock electroporated muscle fiber size recapitulated the results obtained in non-electroporated muscle: TNF significantly reduced the regenerating fiber size if compared with the control (p<0.01 vs. MOCK PBS, by Tukey HSD test), AVP significantly increased the regenerating fiber size, if compared with the control (p<0.01 vs. MOCK PBS, by Tukey HSD test), whereas AVP and TNF combined rescued the TNF-mediated reduction in fiber size (p<0.01 vs. MOCK TNF, by Tukey HSD test) (Fig. 8C). It is noteworthy that TNF did not negatively affect fiber size in muscles overexpressing Hsp70 (p<0.01 vs. MOCK TNF, by Tukey HSD test) (Fig. 8C). Hsp70 overexpression had a slightly hypertrophic effect on the regenerating fiber size, which was not further increased by AVP treatment, either alone or in combination with TNF (Fig. 8C). Taken together, these results demonstrate that TNF-mediated inhibition of regeneration requires Hsp70 downregulation, and suggest that AVP counteracts the TNF-mediated effects on muscle regeneration by maintaining high Hsp70 protein levels.

## Discussion

Inflammatory cytokines play an important role in triggering skeletal muscle fiber growth and regeneration [41], [42]. However, chronic exposure to inflammatory cytokines is deleterious for skeletal muscle homeostasis and function in a variety of pathological conditions [4], [36]. In particular, the negative effects exerted by TNF on muscle include insulin resistance, downregulation of regenerative pathways and upregulation of protein catabolism [4], [5], [43]. There are no effective means of blocking or overriding the effects of TNF on muscle cells besides immunological treatments aimed at TNF [36]. Several reports indicate that insulin-like growth factor I (IGF-I) fails to prevent TNF-mediated muscle atrophy and block of differentiation, one of the reasons being a general downregulation of IGF-I dependent signaling pathways exerted by TNF [44]–[47]. We tested whether intramuscular treatment with AVP, which is also a positive regulator of muscle differentiation, counteracts the negative effects exerted by TNF on muscle. AVP potently induces myogenesis *in vitro* [23], [24], [26], [27], [48]. *In vivo* muscle specific AVP receptor (V1aR) expression is positively modulated upon regeneration (manuscript in preparation). Circulating AVP is increased by exercise, i.e. during intense muscular activity [33]–[35]. This suggests that AVP plays a role in skeletal muscle homeostasis and physiology during postnatal life.

The explorative approach we used to test TNF-AVP cross-talk in muscle cells, which consisted in treating L6 myogenic cells with TNF, AVP, or TNF and AVP combined, showed that AVP completely overrode the TNF blockade of differentiation. This evidence encouraged us to treat regenerating muscle. *In vivo* we found that TNF and AVP displayed opposite effects on regeneration; indeed, TNF delayed and reduced this process, whereas AVP significantly enhanced and extended the kinetics of regenerating fiber formation. As a consequence, AVP had paradoxical effects on regeneration hallmarks: first, neoMHC expression was delayed three days as compared to controls, likely due to prolonged satellite cell proliferation, which in fact gave rise to nascent regenerating fibers for an extended period of time; secondly, the abundance of regenerating fibers of all sizes, including small, newly formed fibers, in the presence of AVP, determined a median regenerating muscle fiber area not significantly different from that of TNF-treated muscle. Regenerating muscle exposed to both TNF and AVP was similar to control muscles, which indicates that it is possible to counteract the negative effects of TNF not only on myogenic differentiation in culture, but also on regeneration. It is well established that satellite cell activation precedes their fusion into regenerating fibers and that persistent Pax7 expression inhibits their progression through myogenic differentiation [49], [50]. In agreement with these reports, our data suggest that: AVP *per se* delays the onset of the differentiation phase of regeneration by enhancing satellite cell activation, still predominant at day 4.5; TNF does not negatively affect satellite cell activation, but it inhibits satellite cell fusion and differentiation, as we and others reported [6], [51]; the combination of AVP+TNF rescues NeoMHC expression at day 4.5 (a phenomenon not observed upon either single treatment) by inducing proper Pax7 downregulation and by favouring the resolution of the inflammatory phase (data not shown). It is worth noting that while inflammatory cytokines are essential for recruitment of satellite cells to muscle fibers [41], pro-myogenic factors, such as IGF-I [52], promote regeneration by accelerating the modulation of inflammatory cytokine effects in regenerating muscles. The full elucidation of the complex signaling pathways triggered by TNF and AVP in muscle is under investigation. However, the abolishment of the inhibitory effect exerted by TNF on muscle regeneration after injury constitutes a tempting approach for the therapy of diseases involving muscle fiber damage [53], [54].

Since most of the adverse effects on patients suffering from muscle disease derive from impaired muscle performance, we were particularly interested in the functional output of regenerating muscle treated with TNF, AVP, or TNF and AVP combined. As mentioned above, these treatments yielded varying degrees of muscle regeneration. When considering physiological muscle outputs, such as tetanic force and fatigue, TNF-treated muscles took markedly longer to functionally recover following injury than muscles subjected to all other treatments. Thus, TNF may affect muscle performance by impairing regeneration. Impaired muscle regeneration may be an additional mechanism in addition to fiber atrophy and sarcomere dismantling [55] accounting for cytokine-mediated decrease of muscle force. TNF treatment not only negatively affected muscle tetanic force but also accelerated muscle fatigue. A TNF-dependent decrease in muscle force and induction of muscle weakness has previously been observed in *ex vivo* experiments [56]. Interestingly, AVP rescued the effects of TNF by increasing the number of regenerating fibers, thus enhancing the tetanic force production and resistance of the regenerating muscles. We found that differences in the fatigue time between treatments inversely correlated with caspase activity, which is in agreement with a previous paper that found an association between increased caspase activation and weakness [57]. The two injections we used in our experimental system did not modify the muscle fiber size of the undamaged, i.e. non-regenerating, fibers (data not shown). On the basis of these findings, we infer that the differences in evoked muscle performance are associated with variations in the degree of muscle regeneration, and not with hypertrophy/atrophy phenomena in the remaining (undamaged) portion of the muscle. The association between the extent of muscle regeneration and its functional output indicates how important prompt and efficient regeneration following injury is if proper muscle function is to be maintained. Worth noting, the control regenerating muscle does not fully recover within two weeks following injury, at which time it still develops a force significantly lower than uninjured muscles. Our observation is in agreement with other reports, showing that the functional gap persists for several weeks following experimentally induced injury [58]. We also noted that control regenerating muscles exert about the same tetanic force at one and two weeks of regeneration, which suggests the occurrence of a prompt recovery following muscle injury (within the first week).Thus repeated injury events, such as those observed in dystrophic muscles, are particularly harmful when occurring within a few days and can lead to complete loss of muscle function.

The evidence, based on our *in vitro* experiments, that AVP and TNF directly modulate myogenic differentiation, led us to investigate the molecular mechanisms underlying this phenomenon. We recently demonstrated that TNF-mediated caspase activation plays a pivotal role in regulating myogenic differentiation *in vitro* as well as muscle regeneration [6], [11]. We further confirmed the regulatory role of caspase activity in regenerating muscle by showing that AVP blocks the TNF-induced increase in caspase activity, thus promoting regeneration. Skeletal muscle regeneration is an additional example of the involvement of caspases in the regulation of embryonic stem and somatic cell differentiation [6], [59]–[61]. Hsp70 and PW1 are two important modulators of caspase activity. PW1 is expressed in stem cells, including myogenic cells, as well as in regenerating muscle fibers [6], [14]. Moreover, it recruits components of the p53-dependent apoptotic pathway to modulate myogenic differentiation in the presence of TNF [11]. *In vivo* PW1 modulates cachexia and fiber size in concert with p53 [13]. Hsp70 plays an anti-apoptotic role through the inhibition of the apoptosome in many cell types [17]. We discovered, by two-dimensional electrophoretic analysis and liquid chromatography coupled with tandem mass spectrometry, that Hsp70 binds to PW1. Given the pivotal role played by PW1 in caspase activation in response to TNF, and the role played by Hsp70 as a controller of caspase activation, we hypothesized that their binding might play a role in the regulation of caspase activity by TNF and/or AVP in muscle cells. We demonstrated that PW1 and Hsp70 are expressed and colocalize in myogenic cells in both growth and differentiation conditions. Moreover, we showed, by means of reciprocal immunoprecipitation experiments, that Hsp70 binds to PW1 in L6 cells. PW1-Hsp70 binding holds true in non-muscle cells, where ectopically expressed PW1 is co-immunoprecipitated with endogenous Hsp70. This suggests that Hsp70 inhibits caspase activation in response to TNF by binding PW1 and blocking its ability to elicit caspase activation. Indeed, we found that upregulation of Hsp70 expression by various means reduces TNF-mediated caspase activation both *in vitro* and *in vivo*: we overexpressed Hsp70 in L6 cells and found that Hsp70 overexpression reduces the number of cells with active caspases in the presence of TNF; *in vivo* Hsp70 is expressed in skeletal muscle and its expression levels inversely correlate with caspase activity in muscles treated with TNF and/or AVP. Interestingly, Hsp70-overexpressing transgenic mice are resistant to age-related muscle functional deficits [18], Hsp70 attenuates skeletal muscle damage induced by cryolesions [19] and cardiac tissue is protected by Hsp70 from myocardial ischemic injury [62]. In addition, Hsp70 induction by heat stress or pharmacological treatment facilitates the regeneration of injured skeletal muscle [63], [64] and increases the survival of transplanted myoblasts [65]. For all these reasons, Hsp70 appears to be an important factor in tissue protection and recovery following stress or damage. We found that Hsp70 protein levels correlate with the regenerative capacity of injured muscles, which highlights a parallelism between caspase activity repression and induction of regeneration by Hsp70. It is noteworthy that TNF and AVP exerted opposite effects on Hsp70 protein levels but not on PW1 protein levels in regenerating muscles. Hsp70 overexpression *in vivo* overrode the inhibitory effect of TNF on muscle regeneration, indicating that Hsp70 downregulation negatively modulates regeneration. Interestingly, AVP increased endogenous Hsp70 protein levels and enhanced regeneration in the absence or presence of TNF. AVP and Hsp70 overexpression (by electroporation-mediated gene delivery) appeared not to be synergistic on the regenerating fiber size, thereby suggesting that either treatment on its own reaches an Hsp70 expression threshold that triggers the maximal muscle regeneration effect.

**Figure 9:**
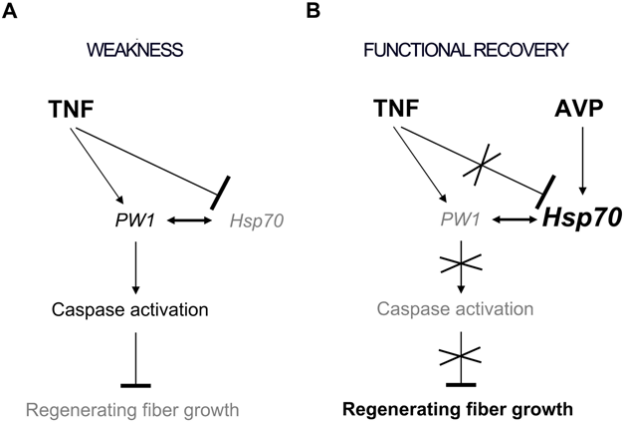
Model for the proposed mechanism underlying the differential effects exerted by TNF and AVP on skeletal muscle regeneration and function. A) TNF enhances caspase activation in regenerating muscle through PW1 and concomitant downregulation of Hsp70 expression. The latter binds to PW1 and diminishes caspase activation in myogenic cells. The reduced extent of regeneration and the delayed fiber growth result in poor muscle performance. B) When combined with TNF, AVP increases Hsp70 expression, thereby yielding a caspase activity level that is comparable to that of the control. This determines an abundant formation and growth of regenerating fibers, which in turn results in a recovered functional output of the injured muscle.

In conclusion, we show that it is possible to counteract the negative effects of TNF on both muscle regeneration and performance by AVP treatment *in vivo*. This occurs through previously unidentified cross-talk between TNF and AVP pathways that is based on Hsp70 protein levels (summarized in Fig. 9). Hsp70 is an important modulator of muscle regeneration and its overexpression counteracts the TNF-dependent inhibition of myogenesis *in vivo* responsible for hampering muscle recovery and performance. We have previously shown that TNF-mediated muscle wasting is associated with defective muscle regeneration [4]. TNF-mediated caspase activation is a prerequisite for muscle regeneration inhibition [6], and Hsp70 may counteract this block of muscle regeneration by inhibiting caspase activation in muscle cells. The negative effects exerted by TNF on muscle regeneration can be rescued by hormonal (AVP), pharmacological (caspase inhibitors) or gene delivery (ΔPW1 [11] or Hsp70 expression) treatments, all of which are aimed at inhibiting caspase activation.

## Materials and Methods

### Cell cultures

The L6 myogenic cell line [66], subclone L6-C5 (henceforth referred to as L6), was cultured as described by Nervi *et al.* [24]. The day after plating, the cultures were differentiated by shifting the medium to DMEM supplemented with 1% FBS and treated with AVP 10^−7^ M (Sigma, St. Louis, MO), TNF 5 ng/ml (Roche Molecular Biochemicals-Boehringer Mannheim), or AVP and TNF combined. PBS was used as a control and the media were replenished every two days. Heat shock was performed by 1 h incubation of L6 cells at 42°C followed by 1 h at 37°C.

### Animals

To induce freeze injury, a steel probe pre-cooled in dry ice was applied to the *tibialis anterior* muscle belly of anesthetized adult (7–8 weeks old) female CD1 mice for 10 seconds. Mice were treated according to the guidelines of the Institutional Animal Care and Use Committee. The method is described in detail elsewhere [6]. To treat muscles, we injected 25 µl of 5 µg/ml TNF-α (i.e. 7.3 µmoles/muscle), 25 µl of 10^−4^ M AVP (i.e. 25×10^−4^ µmoles/muscle), TNF and AVP combined (25 µl of 5 µg/ml TNF-α+25 µl of 10^−4^ M AVP) or 50 µl of PBS in the injured tibialis anterior, 2 and 4 days following injury.

### DNA delivery by electroporation and transfection

To overexpress Heat Shock Protein 70, we used a construct with cDNA for the inducible form of Hsp70 under the control of the SV40 promoter [67], kindly provided by Dr. Marja Jaattela (Institute of cancer biology, Copenhagen, DK). 4.5 days after freeze injury, 22.5 µg of Hsp70 or pCDNA (MOCK) expression vectors were co-injected in the tibialis anterior with 2.5 µg of SNAP-GFP, used as a transfection marker, followed by electroporation. Electroporation was performed by delivering six electric pulses of 20 V each (three with the anode placed on the front of the injured tibialis anterior, followed by three pulses with the polarity inverted). A pair of 3×5 mm Genepaddle electrodes (BTX, San Diego, CA) placed on each side of the muscle was used.

For the *in vitro* experiments, L6 cells were transfected by the Lipofectamine method following the Manufacturer's instructions. For each 35 mm Petri dish, 6 µg of a DNA mix, containing Hsp70 (MJ3) or pCDNA (MJ2) [67] in a 5∶1 ratio with Ref Fluorescent Protein (dsRED; Clontech, Mountain View, CA) was used. For the caspase *in situ* assay experiments, the DNA mix contained Hsp70 (MJ3) or pCDNA (MJ2) in a 5∶1 ratio with a SNAP-GFP construct, courtesy of Dr. Tullio Pozzan. Cells were allowed 24 h to express the constructs before the caspase assay was performed.

### Immunohistochemistry

L6 cultures were fixed in 4% formaldehyde buffered solution and permeabilized with methanol. The cells were stained with antibodies against PW1 ( 370 polyclonal Ab, [14]) and Hsp70 (monoclonal, Stressgen, Victoria, BC). Sarcomeric myosin heavy chain staining was performed with the clone MF20 antibody (supernatant, Hybridoma Bank, Iowa University, IO), as described elsewhere [28]. AlexaFluor488-conjugated anti-rabbit (Molecular Probes, Leiden, The Netherlands) and biotin-conjugated anti-mouse with a Cy3-conjugated streptavidin (Jackson Immunoresearch, West Grove, PA) were used as secondary antibodies. Nuclei were counterstained with DAPI. Differentiated L6 cells were identified by sarcomeric myosin heavy chain staining (clone MF20, hybridoma supernatant, Iowa University, IO), as described elsewhere [28].

Photomicrographs were obtained by using an Axioskop 2 plus system equipped with an Axiocam HRc (Zeiss, Oberkochen, GE) at standard 1300×1030 pixel resolution, or a Leica Leitz DMRB microscope fitted with a Leica DFC300FX camera. Quantitative analysis of differentiation was performed by determining the number of nuclei in MF20 positive cells out of the total number of nuclei in at least ten randomly chosen microscopic fields per sample (% differentiation).

### Co-immunoprecipitation and Western Blot analysis

To test for endogenous PW1-Hsp70 interaction *in vivo*, L6-C5 cells were grown to 70% confluence in 150 mm plates and subjected to heat shock treatment before harvesting. The cells were harvested and lysed in 1 ml of lysis buffer (50 mM Tris-HCl, pH 8.0, 5 mM EDTA, 150 mM NaCl2, 0.5% NP-40, and 1 mM PMSF). Nuclear extracts were prepared by centrifugation following sonication. After centrifugation, equal amounts of protein (3 mg for co-immunoprecipitation of endogenous proteins) were immunoprecipitated, using 2 µg of the corresponding antibody. After pre-clearing the extracts with protein A/G (Pierce, Rockford, IL), the PW1 and the Hsp70 proteins were immunoprecipitated using anti-PW1 or anti-Hsp70 antibodies respectively, followed by protein A/G sepharose. The complex was washed three times with lysis buffer and subjected to electrophoresis on a 4–12% gradient SDS-polyacrylamide gel. The protein was transferred to a PVDF membrane, probed with anti-PW1 and anti-Hsp70 antibody, and developed using an ECL chemiluminescence kit (Pierce). 293 cells were grown to 60% confluence in 100 mm plates and transfected with pcDNA3.1, PMT HA-tagged PW1 (exon 9), PEF HA-tagged PW1 (exon 9) or PW1 FL (full-length) by standard calcium-phosphate transfection procedures. Each plate contained pCMV β-galactosidase expression vectors as an internal control and the total amount of plasmid was adjusted with empty pcDNA3.1 expression vector. Cells were harvested 48 h after transfection and lysed in 500 µl of lysis buffer (50 mM Tris-HCl, pH 8.0, 5 mM EDTA, 150 mM NaCl2, 0.5% NP-40, and 1 mM PMSF) per 100 mm plate. After sonication and pre-clearing, the cells lysates were mixed with anti-PW1 antibody, anti-HA antibody (C12A5 SIGMA) or anti-Hsp70 (Stressgen) for two hours, followed by overnight precipitation with protein A/G sepharose (Pierce). For immunoblotting, proteins were resolved in 4–12% SDS-PAGE, then transferred to PVDF membranes and probed with antibodies to PW1 or Hsp70 (Stressgen). Tissue extracts, obtained from muscle at 4.5 days following regeneration [4], were immunoblotted with antibodies to Pax7, desmin (Sigma) or tubulin (Sigma). Immunoblots were developed using horseradish peroxidase-conjugated antibodies, followed by detection with enhanced chemiluminescence.

### Caspase activity assays

The CaspGLOW™ red active caspase staining kit (Biovision, Old Middlefield Way, CA) was used to quantify caspase activity from muscle lysates. The injured area of the tibialis anterior was isolated, finely minced and transferred to 1.5 ml tubes. Samples were then incubated with a Red-VAD-FMK 1∶300 dilution in PBS, in a water bath at 37°C for 45 min. After centrifugation at 150 g, samples were washed twice in the Manufacturer's Wash buffer for 10 min each, and lysed in 1 ml of lysis buffer (5 mM Tris-HCl pH = 8; 10 mM EDTA; 0.5% Triton) for 30 min at 4°C. The cytosolic fraction was used to perform the enzymatic assay, while the nuclear pellet was used to measure DNA content, as described previously [6], [68]. Fluorometric readings were performed at a wavelength pair of 540/570 nm excitation/emission for caspase activity, and 365/460 nm excitation/emission for DNA content. Samples were normalized by DNA content and expressed as the fold increase over controls (injured, PBS-treated muscle).

Alternatively, CaspGLOW™ was used for an *in situ* caspase assay on myogenic cells. Apoptosis was induced in L6 cells by overnight treatment with 2.5 µg/ml puromycin (Sigma, St. Louis, MO) [69]. Alternatively, caspase activation was triggered by 5 ng/ml TNF treatment overnight in GM. The adherent cells were detached by digestion with trypsin and pooled with the floating cells, and the assay was performed following the Manufacturer's instructions for cells in suspension.

### RT-PCR

Total RNA was prepared from the tibialis anterior using Trizol Reagent (Invitrogen, Carlsbad, CA), following the manufacturer's protocol. RT-PCR was performed using 2 µg of total RNA reverse-transcribed using Moloney murine leukemia virus reverse transcriptase (M-MLV RT; Invitrogen). PCR reactions were carried out in a final volume of 50 µl in a buffer containing 1 µl of RT reaction, 200 µM dNTP, 1.5 mM MgCl2, 0.2 µM of each primer, and 1 U of Taq-DNA polymerase (Invitrogen). The PCR products were analyzed in 2% agarose gel. The following specific primers were used:

Neonatal MHC: forward: 5′-AACTGAGGAAGACCGCAAGAATG-3′, reverse: AAGTAAACCCAGAGAGGCAAGTGACC-3′.

Glyceraldehyde 3-phosphate dehydrogenase transcript (used as internal control): GAPDH: forward: 5′-AACATCAAATGGGGTGAGGCC-3′, reverse: 5′-GTTGTCATGGATGACCTTGGC-3′.

### Functional analysis

Functional analysis was performed according to a previously described protocol [70], [71]. Briefly, the tibialis anterior was carefully dissected, the mass measured (*weight*) and vertically mounted in a temperature controlled chamber (30°C), where it was immersed in a standard Krebs-Ringer bicarbonate buffer solution (Sigma) and continuously gassed with a mixture of 95% O_2_ and 5% CO_2_. One end of the muscle was linked to a fixed clamp while the other end was connected to the lever-arm of an ASI 300 b Dual-Mode actuator/transducer with a non-compliant nylon wire. Muscles were electrically stimulated by means of a pair of electrodes, and evoked forces were continuously acquired. Muscles were initially stimulated with three 0.5 ms single pulses and subsequently with two trains of 0.1 ms pulses performed at an unfused frequency (120 Hz pulses for 0.4 s). To evoke tetanic force (*tetanic force*), muscles were then stimulated with two trains of 0.1 ms pulses (160 Hz pulses for 0.4 s). *Specific force* was calculated by dividing the tetanic force by the mass of each muscle. Finally, muscles were subjected to a series of closer trains of pulses (0.4 s train of 120 Hz pulses) to induce isometric fatigue (*fatigue index*). During the fatigue stimulation, we measured the ability of the muscle to resist to repeated stimulations by calculating the time required to halve the value of its own maximum force (*fatigue time*).

### Morphometric analysis and statistics

For the morphological evaluation of skeletal muscle, cryosections were stained with Haematoxylin and Eosin (H&E, Sigma) using standard methods. Morphometric evaluation of the sections was carried out as described previously [6]. Briefly, photomicrographs of all the regenerating, H&E stained fibers (identified by morphological criteria, i.e. centrally located nuclei) in any cryosections were taken at standard (1300×1030 pixel) resolution and analyzed using Scion Image software (version Beta 4.0.2, Scion Corporation, Frederick; MD). For the evaluation of fiber size, between 200 and 1000 cross-sectioned fibers per sample were analyzed. Quantitative data were obtained from at least three independent experiments in triplicate and the values are expressed as mean±SEM.

To statistically analyze the muscle performance as a function of the various treatments used in this study, we used Principal Component Analysis (PCA). PCA is a procedure for analyzing multivariate data that establishes “metric” differences between groups under investigation on the basis of their characterizing multivariate data [72]. We studied 6 treatment groups, each of which contained numerous individual samples. Several parameters that are indicative of muscle functional properties (maximum force, weight of each muscle, specific force, fatigue index and fatigue time) were considered in the PCA, as previously described [72]–[74]. PCA transforms the original variables into new uncorrelated ones that can be displayed in a bidimensional space and that account for most of the variability observed, thus reducing the dimensionality of the data and allowing a large number of variables to be visualized in a two dimensional plot. The coefficients by which the variables are multiplied to obtain the 1^st^ and 2^nd^ principal components are shown in Table 3. The highest score coefficients indicate the variables that correlate most closely with the two principal components. A diagram of the values obtained from the muscles in each group was plotted in the bidimensional space, defined by the 1^st^ and 2^nd^ principal component functions The use of the 1^st^ and 2^nd^ principal components allows the groups being investigated to be effectively discriminated.

To compare the centroids denoted by the groups, a Manova (multivariate analysis of variance) was performed on the variables. One-way or two-way Anova were used respectively for one or two variate analysis. Either the least squares difference (LSD) test or the Tukey LSD test was used for the *post hoc* comparison between specific groups. The significance level was set at a p<0.05, if not otherwise specified. Statistical analyses were performed with SPSS (statistical package for social sciences) 9.0.

## Supporting Information

Figure S1 Analysis of the PW1 immunoprecipitation products by two-dimensional electrophoretic analysis and liquid chromatography coupled with tandem mass spectrometry (LC-MS/MS) in two mammalian cell lines. Endogenous PW1 was immunoprecipitated from two mouse myogenic cell lines, F3 and C2C12. Murine PW1 was immunoprecipitated following forced expression in human HEK293 cells. Immunoprecipitation products were analyzed by two-dimensional electrophoretic analysis and liquid chromatography coupled with tandem mass spectrometry (LC-MS/MS). The spots analyzed further were chosen after a Progenesis analysis (Progenesis software was used to vectorize the spots in gels and to identify differently expressed spots among the samples. 43 spots were picked, digested overnight and analyzed by LC-MS/MS). F3 was used as a negative control because these cells do not express PW1, and spots present in F3 were consequently considered as unspecific binding. Hsp70 and VDAC-1 appeared as potential PW1 binding partners of both endogenous and overexpressed PW1, while HNRP and enolase were detected only when PW1 was overexpressed.
(0.98 MB TIF)
