# Supplementary figures and images for: Modulation of Caspase Activity Regulates Skeletal Muscle Regeneration and Function in Response to Vasopressin and Tumor Necrosis Factor
Source: PLoS One. 2009 May 18;4(5):e5570. doi: 10.1371/journal.pone.0005570 (PMC2680623; doi:10.1371/journal.pone.0005570)

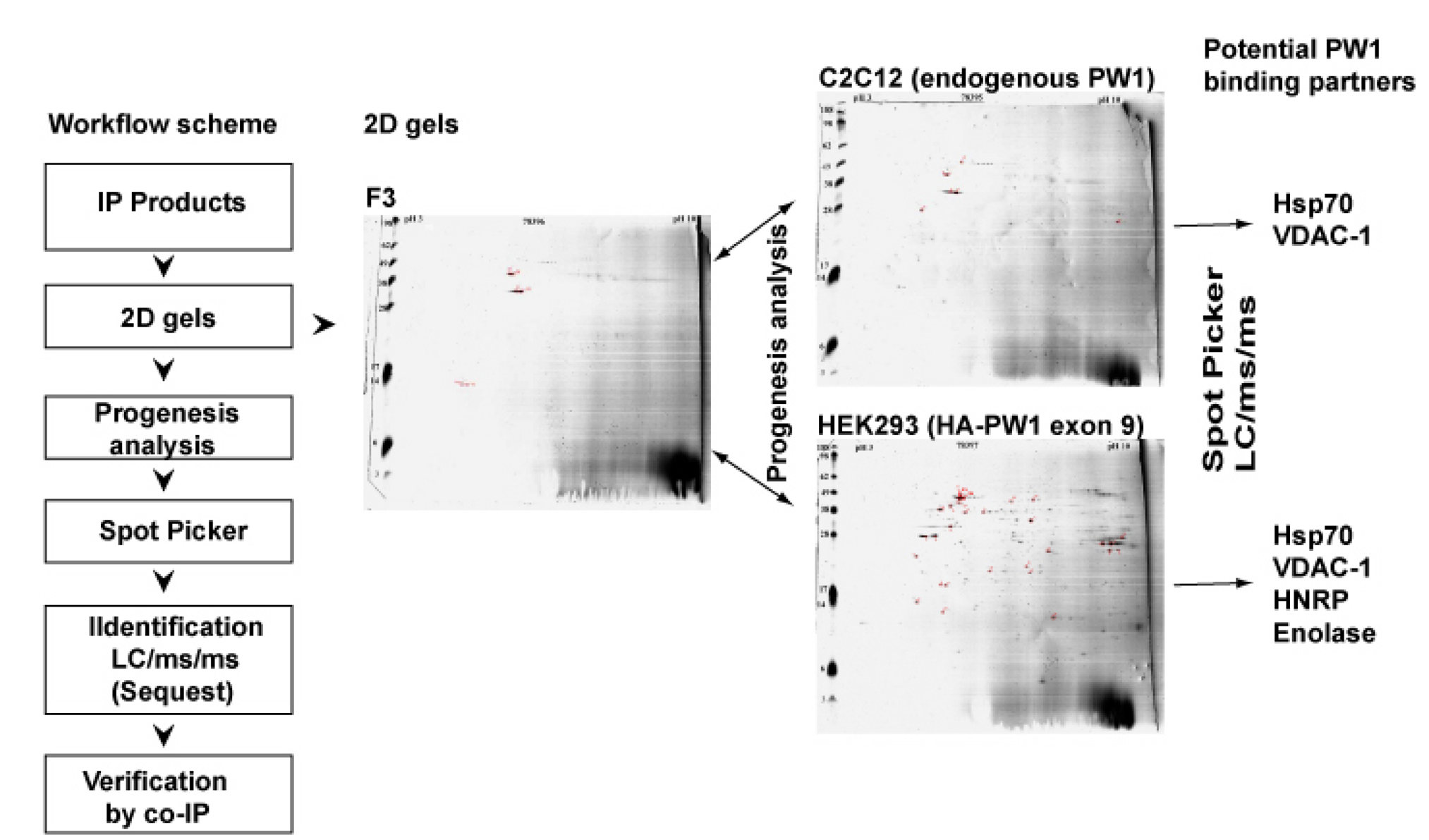

Supplement: Figure S1 — Analysis of the PW1 immunoprecipitation products by two-dimensional electrophoretic analysis and liquid chromatography coupled with tandem mass spectrometry (LC-MS/MS) in two mammalian cell lines. Endogenous PW1 was immunoprecipitated from two mouse myogenic cell lines, F3 and C2C12. Murine PW1 was immunoprecipitated following forced expression in human HEK293 cells. Immunoprecipitation products were analyzed by two-dimensional electrophoretic analysis and liquid chromatography coupled with tandem mass spectrometry (LC-MS/MS). The spots analyzed further were chosen after a Progenesis analysis (Progenesis software was used to vectorize the spots in gels and to identify differently expressed spots among the samples. 43 spots were picked, digested overnight and analyzed by LC-MS/MS). F3 was used as a negative control because these cells do not express PW1, and spots present in F3 were consequently considered as unspecific binding. Hsp70 and VDAC-1 appeared as potential PW1 binding partners of both endogenous and overexpressed PW1, while HNRP and enolase were detected only when PW1 was overexpressed. (0.98 MB TIF) [file pone.0005570.s001.tif]
